# Changes in Physical Activity and Psychological Variables Following a Web-Based Motivational Interviewing Intervention: Pilot Study

**DOI:** 10.2196/resprot.4623

**Published:** 2015-10-29

**Authors:** Sasha L Karnes, Barbara B Meyer, Lisa M Berger, Michael J Brondino

**Affiliations:** ^1^ University of Wisconsin--Whitewater Department of Psychology Whitewater, WI United States; ^2^ University of Wisconsin--Milwaukee Department of Kinesiology Milwaukee, WI United States; ^3^ University of Wisconsin--Milwaukee Helen Bader School of Social Welfare Milwaukee, WI United States

**Keywords:** motivational interviewing, physical activity, adults, Web-based, intervention, health care, psychology

## Abstract

**Background:**

Web-based interventions for enhancing physical activity participation are in demand for application in health care settings. Recent research suggests Web-based interventions that are based on motivational interviewing are effective to increase physical activity. It is unclear whether motivational interviewing can influence targeted psychological variables such as perceived readiness, willingness, and ability to participate in physical activity.

**Objective:**

The aims of this study were to determine whether there were changes in physical activity and psychological variables associated with readiness, willingness, and perceived ability to participate in physical activity following completion of a novel Web-based intervention. The goal of the motivational interviewing–based intervention was to increase physical activity.

**Methods:**

Twenty-three underactive or inactive urban dwelling adults were recruited at a medical office for participation in a 4-session Web-based intervention lasting approximately 15 minutes per week. Sessions were based on principles of motivational interviewing. Assessment of physical activity was conducted using pedometers immediately prior to intervention participation (pre) and immediately post intervention (post1). Self-report assessments of physical activity and psychological variables were conducted using online surveys at pre, post1, and again at one month following intervention participation (post2).

**Results:**

Comparisons of pre and post1 pedometer recordings revealed significant increases in steps per day (*t*_22_=2.09, *P=*.049). There were also significant changes in total physical activity energy expenditure per week (χ^2^_2_=8.4, *P*=.02) and in moderate intensity physical activity energy expenditure per week (χ^2^_2_=13.9, *P<*.001) over time following participation in the Web-based intervention. Significant changes in psychological variables following participation in the Web-based intervention included: (1) change in stage classification over time (χ^2^_2_=21.5, *P*<.001), where the percentage of participants classified in the action or maintenance stages of change in physical activity increased over time (pre=25% [6/24], post1=71% [17/24], post2=68% [15/22]); (2) decreases in self-reported decisional balance cons (*F*_2,42_=12.76, *P*<.001); (3) increases in self-reported decisional balance pros (*F*_2,42_=16.19, *P*<.001); (4) increases in physical activity enjoyment (*F*_2,20_=3.85, *P*=.04); and (5) increases in self-efficacy (*F*_2,42_=3.30, *P*=.047).

**Conclusions:**

The Web-based intervention piloted in this study shows preliminary promise as a tool to promote physical activity in health care settings. Additional research is needed to test the effectiveness of motivational interviewing compared to a control condition and to refine content by considering mediation by psychological variables in a larger sample.

## Introduction

The United States is experiencing a public health crisis because most adults are not getting sufficient physical activity to promote health and prevent disease [1,2]. Interventions to increase physical activity that are effective and feasible to implement in health care settings are needed. One barrier to feasibility is that health care providers have limited face-to-face time with patients to discuss strategies for increasing physical activity [3]. Even when time allows, approaches such as direct advice-giving are often implemented and unfortunately are not likely to be effective [4]. To mitigate difficulties with lack of time, health care providers and patients alike may benefit from the availability of a Web-based intervention to which patients can be referred. While Web-based interventions are becoming more popular, there is not yet consensus on the specific framework of such interventions for increasing physical activity [5].

Motivational interviewing is one clinical technique [6] with preliminary support as a framework for Web-based delivery of interventions geared at increasing physical activity [7,8]. Motivational interviewing is thought to influence behavior change by enhancing a person’s readiness, willingness, and perceived ability to engage in a particular behavior. The ready, willing, and able constructs of motivational interviewing map onto psychological variables. Psychological variables of interest specifically include readiness to change, decisional balance, enjoyment, and self-efficacy. Although readiness, willingness, and perceived ability are targeted during interventions that are based in motivational interviewing, research is needed to determine if this influences targeted psychological variables.

In summary, it has been established that interventions to increase physical activity are needed and that Web-based delivery may be desirable to address time limitations in health care settings. Although motivational interviewing-based interventions have shown preliminary support for increasing physical activity [4], there is limited research into whether Web-based motivational interviewing is effective for increasing physical activity when offered by health care providers. There is also limited research into the possible changes in psychological variables related to participating in motivational interviewing. Understanding whether interventions based on this method do in fact change psychological variables could provide valuable information for determining what specific content should be included in future iterations of interventions. Therefore, the aims of this study were to determine whether there were changes in physical activity as well as changes in psychological variables associated with readiness, willingness, and perceived ability following introduction to a novel Web-based intervention. The intervention was geared at increasing physical activity and was based on motivational interviewing. The specific aims of this study were to assess whether there were increases in physical activity and changes in targeted psychological variables following participation in the intervention.

## Methods

### Study Design

A repeated measures design was used to conduct a pilot assessment of whether there were changes in physical activity and psychological variables following participation in a Web-based intervention based on motivational interviewing. Data for both physical activity and psychological variables were gathered before intervention participation (pre), immediately after intervention participation (post1) and one month following intervention participation (post2). Links for intervention sessions were emailed to participants once per week for 4 weeks, and each session took approximately 15 minutes to complete.

### Recruitment

Patients at an urban health care clinic in Milwaukee, Wisconsin, were recruited to participate. Flyers advertising weight management were placed at reception desks and circulated in waiting rooms. Patients who were interested provided contact information including personal and physician email addresses to the receptionists. Along with providing contact information, patients were asked whether they met the 2007 Centers for Disease Control and Prevention (US Department of Health and Human Services) guidelines for physical activity. Using the contact information provided, study staff sent email inquiries to the patients’ physicians to obtain medical clearance for participation. In order to be eligible for participation, patients had to self-report insufficient physical activity, have a working email address, and have been cleared for participation by their medical doctor. Eligible and willing patients completed an online informed consent process before engaging in data collection and intervention activities.

### Intervention

The intervention was novel and consisted of a Web-based program that was designed based on the principles of motivational interviewing. Each intervention session was self-guided and included prompts for the participant to engage in self-reflection on their physical activity behavior and related thoughts. The specific mechanism by which self-reflection was facilitated was that each session included a set of questions prompting the participant to generate a written response. All of the sessions were presented in black text with a white screen created using Qualtrics survey software. Textbox 1 shows an example of the series of questions included in the first session—questions which were based on motivational interviewing principles of building rapport, creating a discrepancy between current physical activity behaviors and goals, and addressing barriers to physical activity. Note that each question was followed by a text area for the participant to respond and a button to advance to the next screen.

**Examples of questions prompting participant self-reflection.**

- Welcome! Glad you decided to participate! Your doctor talked to you about your physical activity. What are some of your concerns about how much physical activity you are getting?
- Those are certainly valid concerns. Thank you for sharing. Now that you have shared your concerns, let’s consider some of your thoughts about physical activity. What are some reasons that you aren’t getting as much activity as you or your doctor would like?
- Those are difficult challenges to overcome, and you’ve done a great job thinking this through. It really is hard to get as much activity as is recommended. Describe, if you would, a time in the past when you were more active or activities that you were able to do and how those challenges fit in.

Notice that the tone of the questions was such that the participant was greeted with validation and acceptance, consistent with the tone of motivational interviewing. Responses were prompted with the goal of guiding the participant’s thinking in the direction of feeling ready, willing, and able to engage in physical activity. One deviation from traditional motivational interviewing was that the questions were not individually tailored. That said, after the first session participants were sent a personalized email message thanking them for participation and reflecting on their responses. This email message was the only tailored component of the intervention and was implemented to facilitate participant retention.

Sessions two through four followed the same format and delivery style as the first session. During the second session, content included self-reflection on feedback about physical activity and was geared toward eliciting a commitment to change physical activity behaviors. The third session involved creation of a change plan and was geared at strengthening the commitment to change. During the fourth and final session, efforts to change were affirmed, and commitment to change was further reinforced.

### Measures

#### Overview

Demographic information was gathered using an online self-report inventory at pre. Online self-report instruments were also used to gather subjective reports of physical activity and psychological variables at pre, post1, and post2. Pedometer recordings were gathered by the participant and emailed to the researchers at pre and post1. To avoid the potential confounding influences of having participants monitor steps, participants were instructed not wear their pedometers or record steps during the intervention phase.

#### Physical Activity

Assessment of physical activity was conducted using both pedometers and self-report surveys. Using pedometers is recommended to accurately capture total amounts of physical activity among adults, given the likelihood of underestimation of physical activity during self-reports [9]. As such, Yamax SW-200 pedometers were used to measure steps taken per day. Total steps over the course of 7 days was recorded with the pedometer worn at the hip. Each participant was given instructions for recording and emailing daily step-counts to the principal investigator. Pedometer data were compiled by tabulating the total number of steps recorded by the participant each day for 7 days. Yamax pedometers have previously demonstrated a high degree of reliability given estimates of 100% consistency in counting steps when compared to hand-tallied observed step-counts [10].

For the self-report tool, the International Physical Activity Questionnaire (IPAQ) short form for self-administration was used to assess the number of minutes spent participating in physical activity in the past 7 days [11]. The participants were asked to report number of days per week and hours and minutes of engagement in behaviors classified as vigorous, moderate, walking, and sitting. Total physical activity metabolic equivalent of task minutes (METs) per week were calculated in accordance with IPAQ scoring guidelines by summing the following: (1) 3.3 × walking minutes × walking days, (2) 4.0 × moderate intensity activity minutes × moderate days, and (3) 8.0 × vigorous intensity activity minutes × vigorous intensity days. The results of prior research indicate that the IPAQ has sufficient test-retest reliability (*r*=.66-.88, 95% CI 0.73-0.77) and moderate criterion validity against accelerometers when measuring moderate intensity physical activity among adults (*r*=.46-.81, 95% CI 0.23-0.36) [11].

#### Psychological Variables

Readiness

Readiness to engage in physical activity was assessed by examining participant stage classification using the Exercise Stages of Change Questionnaire [12]. Individuals were categorized in stages precontemplation, contemplation, preparation, action, or maintenance, where higher stages such as action and maintenance reflect more engagement in physical activity compared to lower stages. The Exercise Stages of Change Questionnaire showed modest significant correlations with step recordings at pre (*r*=.38, *P*=.02) and post1 (*r*=.47, *P*=.03). The Exercise Stages of Change Questionnaire has previously shown high test-retest reliability among adult men and women over a two-week period with a kappa index of .78 [13].

Willingness

Willingness to engage in physical activity was assessed by examining both decisional balance related to physical activity and physical activity enjoyment. A decisional balance questionnaire [14] was used to ask participants to rate the importance of statements pertaining to making decisions about whether to engage physical activity. Greater endorsement of exercise pros and lower endorsement of exercise cons would suggest greater likelihood of engaging in physical activity. In this study, the decisional balance questionnaire demonstrated internal consistency reliabilities that were acceptable to high for pros at pre (*r*=.62), post1 (*r*=.39), and post2 (*r*=.94) as well as cons at pre (*r*=.72), post1 (*r*=.81), and post2 (*r*=.76).

Higher scores on the Physical Activity Enjoyment Scale (PACES) [15] indicated greater enjoyment. Internal consistency ratings were high in the study at pre (*r*=.96), post1 (*r*=.93), and post2 (*r*=.94), and the scale previously demonstrated discriminant validity when correlated with boredom proneness among adults during exercise (*r*=−.30*, P*<.05) among adults [16].

Perceived Ability

Perceived ability to engage in physical activity was assessed using a self-efficacy questionnaire [17] where higher scores indicated greater perceived ability. Internal consistency ratings were good in the study at pre (*r*=.74), post1 (*r*=.81), and post2 (*r*=.74).

### Statistical Analysis

Step-count data were normally distributed and therefore paired samples *t* tests were completed to compare average steps per day from pre to post1. Due to positive skew of IPAQ data, Friedman nonparametric tests with post hoc Wilcoxon analyses were conducted to assess changes in self-reported physical activity over time. Friedman and Wilcoxon signed-rank follow-up tests were also conducted to assess changes in stage classification over time. Since decisional balance and self-efficacy data were normally distributed, a series of repeated measures analyses of variance (ANOVAs) was completed to assess for change over time. Enjoyment data violated the sphericity assumption; therefore the multivariate test results including Wilks lambda values were interpreted from the repeated measures ANOVAs. Analyses were conducted using SPSS version 22.0 statistical software (IBM Corporation).

## Results

### Participant Characteristics

**Figure 1:**
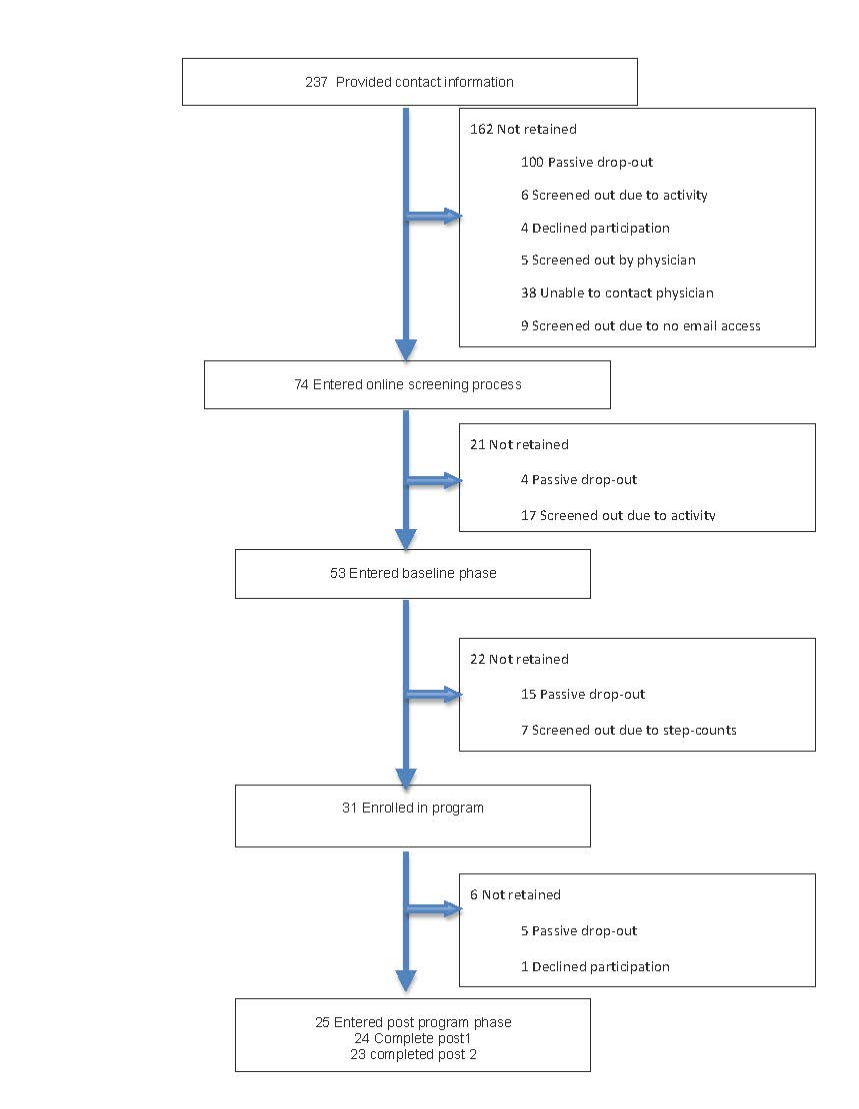
Recruitment, enrollment, and retention.

Among the 237 persons who responded to the recruitment flyer, 31 enrolled in the intervention, and ultimately 23 participants (87%, 20/23, female; mean age 46, SD 11.0) completed all assessments and intervention sessions. See Figure 1 for a flow chart of recruitment, enrollment, and retention. The cohort of participants who completed the study was racially diverse, with 44% (10/23) of the participants identifying as other than white. The majority of the participants 96% (22/23) reported being overweight or obese.

### Changes in Physical Activity

Significant increases were found for average daily steps from 5252.99 (SD 2412.66) at pre to 6425.05 (SD 2783.73) at post1 (*t*_22_ *=*2.09 *P=*.049), total weekly energy expenditure was 1918.33 METS at pre (SD 2755.11) to 3457.12 METS at post2 (SD 5670.63) (χ^2^_2_=8.4 *P*=.02), and moderate weekly energy expenditure 218.00 at pre (SD 417.15) to 1091.00 (SD 2589.88) at post2 (χ^2^_2_=13.9, *P<*.001).

### Changes in Psychological Variables

Stage classification percentages changed significantly over time (χ^2^_2_ =21.5, *P*<.001) and are detailed in Table 1. Mean ranks increased from pre (1.39) to post1 (2.23) and post2 (2.39). Follow-up testing revealed significant changes from pre to post1 (*z=*3.02 *P*=.00 and pre to post2 (*z=*3.37, *P*<.001) but not post1 to post2 (*z*=0.79, *P*=.43). Cross-tabulation results (see Table 1) further reveal that while there were no participants in the precontemplation stage at any point, the percentage of participants in the contemplation stage was lower at post1 and post2 compared to pre, whereas more participants were in the action and maintenance phases at post1 and post2 compared to pre.

**Table 1:** Percentage of participants in stage classifications pre, post1, and post2.

	Pre (n=24) n (%)	Post1 (n=24) n (%)	Post2 (n=22) n (%)
Contemplation	15(63)	7 (29)	5 (23)
Preparation	3 (13)	0	2 (9)
Action	5 (21)	13 (54)	8 (36)
Maintenance	1 (4)	4 (17)	7 (32)

Sources of variance, *F* ratios, and significance values for the repeated measures ANOVA detailing significant changes over time in psychological variables associated with willingness and perceived ability are provided in Table 2. There were significant changes in directions for decisional balance pros, decisional balance cons, physical activity enjoyment, and self-efficacy. Notably, these changes were in the expected direction given that relevant to physical activity, participants self-reported increases in decisional balance, enjoyment, and self-efficacy over time.

**Table 2:** Repeated measures ANOVA source table for psychological variables over time.

Source		Degrees of freedom	Sum of squares	Mean sum of squares	F statistic	P value
**Decisional balance, pros** **(n=22)**						
	Between groups	2	654.21	327.11	16.19	<.001
	Within groups	42	848.46	20.20	—	—
**Decisional balance, cons** **(n=22)**						
	Between groups	2	357.21	178.61	12.76	<.001
	Within groups	42	588.12	14.00	—	—
**PACES (n=22)** ^a^						
	Between groups	2	—	—	3.85	.04
	Within groups	20	—	—	—	—
**Self-efficacy** **(n=21)**						
	Between groups	2	161.85	80.92	3.30	.047
	Within groups	42	1030.82	24.54	—	—

^a^Wilks lambda values reported due to sphericity assumption violation.

##  Discussion

### Principal Findings

Overall, results of the pilot study were promising regarding effectiveness of the Web-based intervention tested. There were changes in physical activity as well as changes in psychological variables associated with readiness, willingness, and perceived ability to participate following participation in the motivational interviewing–based intervention. Changes were evident based on significant increases in both step-counts from pre to post1 and self-reported moderate and total weekly physical activity. Changes in psychological variables over time included significant increases in physical activity–related stage classification, decisional balance, enjoyment, and self-efficacy. One plausible explanation for the observed increases in physical activity is that changes in psychological variables (ie, readiness, willingness, perceived ability) contributed to enhanced participation in physical activity. While the changes in psychological variables may have come about due to the direct impact of the motivational interviewing–based intervention, this assertion cannot be fully supported without further research. Nonetheless, preliminary findings are encouraging.

Results regarding physical activity and psychological variables are particularly meaningful given the strengths of the current study. With regard to assessment of physical activity, the use of both pedometers and self-report surveys builds confidence that observed changes were valid [10]. Further, although the sample size was small, the sample was racially diverse and likely representative of the population to which the intervention would apply. That is, the sample included adult patients at an urban health care clinic who were not engaging in sufficient physical activity. Although patients who were overweight or obese were not explicitly targeted for recruitment, the sample comprised individuals who could benefit from engaging in additional physical activity due to being classified as overweight or obese.

In addition to testing effectiveness using triangulated methods to assess physical activity in a relevant sample, the study examined changes in psychological variables (variables that have not been included in prior research). That is, research on changes in psychological variables targeted during interventions that are Web-based and are based on motivational interviewing was not previously available. Acknowledging changes in targeted psychological variables is critical for understanding the mechanisms by which the intervention is likely to impact physical activity.

### Limitations

The limitations discussed should be taken into consideration when interpreting the results of this study and when planning future research. Due to lack of a control group, causal impact of the intervention cannot be determined. Nonetheless, because changes in physical activity and psychological variables at post1 and post2 are in the expected direction and are in agreement over time, the pilot results serve as a basis for supporting further research. To address this limitation, future research efforts should include a control condition.

Recruitment of a larger sample is necessary to allow sufficient statistical power for comparing the intervention condition to a control condition. Sample characteristics in the current study also reveal greater participation by women who were overweight or obese. In order to determine whether selective drop-out by men or persons of healthy body weight occurred, systematic investigation of those individuals who enrolled but did not follow through with intervention participation may be warranted.

Interpretation of stage classification outcomes is complicated by potential validity issues in the study. Validity concerns specifically pertain to questionable accuracy of stage classification given that more participants self-reported advancing to the maintenance stage (which necessitates 6 months of behavior change) at post2 than is logical or possible when taking into account that only 2 months had elapsed since pre. Because of questionable validity of the stage of change outcomes, continued triangulation with other sources of physical activity data is recommended.

### Conclusions

Results are consistent with previous research, in that they provide further support that Web-based interventions based in motivational interviewing show promise for increasing physical activity participation [7,8]. The Web-based motivational interviewing intervention piloted in the study demonstrates preliminary evidence of effectiveness for enhancing physical activity in a health care setting. The mechanisms by which the motivational interviewing-based interventions impact physical activity may be through changes in targeted psychological variables. Collectively, these findings support the need for additional investigation into the effectiveness and mechanisms of the intervention that was piloted. Specifically, a meditational model should be tested to determine if the intervention directly influences psychological variables and whether changes in psychological variables indirectly contribute to increases in physical activity.

The intervention tested was created to meet the demands of time-limited physicians and their patients. The findings indicate that this intervention may in fact be valuable for health care delivery given that the intervention was effective for increasing physical activity in a health care setting. Benefits of the intervention are that it was very brief and the delivery format required limited involvement of health care professionals. Specifically, the intervention sessions required approximately 15 minutes of participation per week for a total of four weeks, and delivery of the intervention required only emailing links to the participants each week, with one follow-up email provided after the first session. The tasks associated with intervention delivery could be conducted by an individual with less training than a physician, thereby alleviating burden on physician time and still providing a necessary resource for patients who are not getting sufficient physical activity for health and well-being.

## Abbreviations

IPAQ: International Physical Activity Questionnaire
MET: metabolic equivalent of task
PACES: Physical Activity Enjoyment Scale
Pre: prior to intervention participation
Post1: immediately following intervention participation
Post2: one month following intervention participation
